# The Characteristics of AOM and Formation of DBPs: The Role of Molecular Weights and Hydrophobicity

**DOI:** 10.3390/toxics14040349

**Published:** 2026-04-21

**Authors:** Lingfei Ma, Haipu Li, Zhaoguang Yang

**Affiliations:** 1 School of Petroleum Engineering and Environment Engineering, Yanan University, Yanan 716000, China; 2 Center for Environment and Water Resources, College of Chemistry and Chemical Engineering, Central South University, Changsha 410083, China; lihaipu@csu.edu.cn (H.L.); yang@csu.edu.cn (Z.Y.)

**Keywords:** characteristic, AOM, excitation–emission matrix fluorescence, disinfection byproducts

## Abstract

This study investigates the impacts of algogenic organic matter (AOM) distribution characteristics, specifically molecular weight (MW) and hydrophobicity, on the formation of disinfection byproducts (DBPs) derived from Microcystis aeruginosa. This study focuses on both extracellular organic matter (EOM) and intracellular organic matter (IOM) and their contributions to DBP formation. AOM was divided into 12 fractions based on MW and hydrophobicity (transphilic, hydrophilic, and hydrophobic fractions). The results reveal that the hydrophobic fraction (HPO) contributes the most to IOM, while low-MW (<1 kDa) and high-MW (>100 kDa) organic matter are the main components of AOM. An analysis of fluorescent species indicates that humic acid-like and fulvic acid-like compounds derived from the hydrophilic fraction (HPI) of EOM and the hydrophobic fraction (HPO) of IOM are the dominant low-MW (<1 kDa) species. Additionally, aromatic proteins derived from HPO in both EOM and IOM are the dominant high-MW (>100 kDa) fluorescent species. This suggests that proteins or polysaccharides are the primary adsorbents on the membrane during ultrafiltration (UF), while the humic acid component is not significantly deposited. Furthermore, this study identifies that the >100 kDa HPO in IOM serves as the main precursor for trichloromethane (TCM), trichloroacetic acid (TCAA), and dichloroacetic acid (DCAA). In EOM, the precursor for the highest TCMFP (63.6 µg/mg-C) is the >100 kDa HPI, while the highest contribution to TCM (21%) is from the >100 kDa HPO. These findings provide crucial information for controlling DBPs derived from AOM through membrane filtration, particularly in eutrophic water environments.

## 1. Introduction

Algal blooms (e.g., *Microcystis aeruginosa*) cause the release of algal organic matter (AOM) during cell growth and lysis into water, including extracellular organic matter (EOM) and intracellular organic matter (IOM). AOM comprises a wide range of compounds with a broad spectrum of molecular weights and hydrophobicity distributions, such as polysaccharides, proteins, lipids, and amino acids, as well as other traceable organic acids, and AOM can react with disinfectants in drinking water resources to produce disinfection byproducts (DBPs) during the disinfection process [1]. DBPs exhibit mutagenesis, genetic toxicity, cytotoxicity, reproductive development toxicity, and carcinogenicity, and they pose a potential risk to humans [2]. To control DBPs, some water treatment technologies, including adsorption, membrane filtration, enhanced coagulation, and photocatalysis, have been adopted or improved to effectively remove DBP precursors in water utilities.

Hydrophobicity and molecular weight (MW) are the basic properties of AOM and are the most important factors determining retention and membrane fouling in filtration [2,3]. Li et al. [4] found that AOM was predominantly composed of HPI and that organic matter with a molecular weight greater than 30,000 accounted for over 40%. The filtration membrane process is widely considered a promising technology for the treatment of drinking water due to its effectiveness in removing particles, colloids, bacteria, and algae [5,6]. Experiments have shown that there are significant differences in removal capability for components with different MWs and hydrophilicities during filtration [7]. Braeken et al. [8] found that high hydrophobicity lowers retention during membrane filtration, but the influence of hydrophobicity decreases if the molecular size increases.

In addition, DBP formation potentials (DBPFPs) are different in different hydrophilic components. Zhou et al. [9] found that DBPFPs followed the order of hydrophobic fraction (HPO) > transphilic fraction (TPI) > hydrophilic fraction (HPI) for EOM. Several studies have reported the effect of MW in organic matter on DBPFPs. Low-molecular-weight (low-MW, <1 kDa) and high-molecular-weight (high-MW) fractions of EOM and IOM contain significant amounts of DBP precursors [10]. However, when studying AOM and its DBPs, few studies simultaneously considered both MW and hydrophilicity. Compared with previous studies, this work provides a more refined fractionation method by combining molecular weight and hydrophobicity. The quantitative contributions of each subfraction to various DBPs are clarified, which helps us to recognize DBP precursors precisely. Moreover, the membrane selection suggestions based on fraction characteristics can support practical DBP control in eutrophic source water treatment.

However, most previous studies investigating the relationship between AOM and disinfection byproducts (DBPs) have employed molecular weight, hydrophobicity, fluorescence characteristics, and infrared spectra as conventional aspects of characterization [4,11]. For instance, Cheng et al. demonstrated a significant correlation between AOM’s fluorescence properties and molecular weight distribution [12]. Liu et al. [2] found that conventional commercial polyaluminum chloride exhibited superior removal efficiency for hydrophobic and hydrophilic substances with molecular weights around 1 kDa compared to high-molecular-weight polychlorides. While fractional fractionation techniques were occasionally adopted, most studies relied on single fractionation methods as auxiliary approaches [13,14]. For example, Zhao et al. [13] investigated the generation potential of DBPs containing hydrophobic components during the coagulation process. Few studies systematically integrated these two critical characteristics by subdividing AOM into refined subcomponents and elucidating their contribution mechanisms to different DBP types [15]. This study innovatively employed a coupled molecular weight–hydrophobicity analysis method to classify AOM into 12 subcomponents, quantitatively assessing the formation potential of carbon-source and nitrogen-source DBPs within each component.

Therefore, classifying AOM into different fractions and investigating their variation trends are important for controlling DBPs. It is vital to isolate AOM into more homogeneous groups based on chemical or physical properties. Among various isolation methods, resin fractionation and membrane filtration are the most frequently used methods [16]. More detailed information regarding the characteristics of DBP precursors derived from AOM can be obtained based on hydrophobicity and molecular weight (MW). Our previous study showed that protein-like and humic-like components were the main sources of DBPs, with a contribution rate of more than 80%. Existing studies also pointed out that humic acid-like compounds have a much higher contribution rate to chlorinated hydrocarbons generated after chlorination than their total organic carbon content fraction, and nitrogen-containing compounds (which belong to protein-like substances) can also produce DBPs after a chlorination reaction. Considering that these fractions contain protein-like and humic-like components, substances that are directly related to DBPs, they need to be further investigated by excitation–emission fluorescence spectroscopy (EEM). Hua et al. [10] identified the strong dependence of IOM-derived haloacetic acids (HAAs) and trihalomethane (THM) on MW properties and fluorescence. And the fluorescent intensity of AOM correlated well with HAA formation potential (HAAFP) [17].

The objectives of this study are to analyze organic fractions with different MWs and hydrophilicities for EOM and IOM, investigate their DBPFPs, and also analyze the characteristics and DBPs of organic fractions. Through this study, we aim to identify the characteristics of THMs and HAA precursors and elucidate the relationship between the characteristics of AOM and DBPs, with a particular focus on molecular weight and hydrophobicity. Ultimately, we intend to propose targeted suggestions for the control of DBPs via membrane filtration.

## 2. Materials and Methods

### 2.1. AOM Sample

The AOM sample used in this study was obtained from Microcystic aeruginosa (Collection No. HB1322) purchased from the Culture Collection of Algae at the Institute of Hydrobiology, Chinese Academy of Sciences, and is one of the most popular blue-green species found in freshwater bloom. Microcystic aeruginosa was cultivated according to our previous culture method. EOM and IOM were also obtained from Microcystic aeruginosa by the previous method.

### 2.2. Hydrophobic/Philic Fractionation

The hydrophobic/philic fractionation of AOM was performed by column chromatography with DAX-8 and DAX-4 resin (Supelite, St. Bellefonte, PA, USA) filled inside the column. The column was made of plexiglass with an inner diameter of 1.0 cm and a height of 20 cm and a void volume of 8 mL. AOM was acidified to pH 2 by sulfuric acid, and then it was passed through DAX-8 resin followed by DAX-4 resin. The effluent from the DAX-4 resin was collected as the HPI. The dissolved organic matter (DOM) fraction absorbed by the DAX-8 resin was subsequently back-eluted from the resin column with 2 bed volumes of eluent. In this study, 2 bed volumes (BV) of sodium hydroxide solution (NaOH) with pH 11 was referred to as the HPO. The DAX-4 resin retained organic compounds comprising TPI, which was also eluted using the same 2 BV NaOH. The flow rate was set to be 8 BV/h to avoid flow-related disturbance to adsorption capacity. The volume of the obtained fractions was adjusted to match the initial sample volume using ultrapure water, and their pH was subsequently adjusted to 7 with sulfuric acid or sodium hydroxide. The detailed hydrophobicity procedure can be found in previous studies [18].

### 2.3. Molecular Weight Fractionation

The molecular weight fractionation of the AOM was performed using ultrafiltration (UF) membranes with molecular weight cut-offs of 100 kDa, 10 kDa, and 1 kDa. The UF procedure involved the continuous ultrafiltration of a 300 mL sample in an ultrafiltration cup with agitation, while the nitrogen pressure was maintained at 50 psi of 300 mL using a 100 kDa membrane, and the permeate was collected first (Figure S1). The volume of the retained solution was then gradually reduced to 60 mL via filtration with the same 100 kDa membrane, after which the ultrafiltration cup was rinsed with ultrapure water to restore the volume to 300 mL. Filtration was continued until the volume decreased to 60 mL once more. This rinsing process was repeated twice more to remove DOM with an MW below the MWCO. The retentate was collected, and its volume was subsequently adjusted to the desired level by dilution with ultrapure water to restore it to the initial loading volume. Each fraction was then diluted to restore its DOC concentration to that of the original initial solution. This cycle was repeated sequentially using the 10 kDa and 1 kDa membranes. This ultrafiltration method yielded four fractions with nominal organic molecular weight (MW) ranges of >100 kDa, 100–10 kDa, 10–1 kDa, and <1 kDa. A detailed membrane fractionation process can be found in previous studies [19].

### 2.4. Chlorination Experiment

All samples were adjusted to pH 7.0 ± 0.2 in amber glass bottles before the chlorination experiment. Disinfection experiments were conducted by adding sodium hypochlorite to each sample at a dose of Cl_2_:DOC = 10 (mmol Cl_2_ mg^−1^ C) followed by the incubation of the samples at room temperature (25 ± 1 °C) in the dark. The samples were quenched with sodium thiosulfate after 3 d. After chlorination, the total chlorine residuals and free chlorine residuals in samples were determined, and a portion of the sample was used to determine the concentration of DBPs.

### 2.5. Analytical Methods

#### 2.5.1. Determination of DBPs

Trichloromethane (TCM), dichloroacetonitrile (DCAN), trichloroacetonitrile (TCAN), trichloronitromethane (TCNM), 1,1-dichloro-2-propanone (DCP), chloral hydrate (CH), 1,1,1-trichloro-2-propanone (TCP), and two haloacetic acids including trichloroacetic acid (TCAA) and dichloroacetic acid (DCAA) were detected by a gas chromatograph with an electron capture detector (Agilent, St. Clara, CA, USA). The column used was an HP-5 column (60 m × 0.25 mm × 0.25 μm,), which was used based on the methods of other literature [10].

#### 2.5.2. DOC

A total organic carbon analyzer (TOC-VCPH, Shimadzu, Kyoto, Japan) was used to determine the DOC concentrations of samples, with potassium hydrogen phthalate, anhydrous sodium carbonate, and sodium hydrogen carbonate used as standards. The measurement range of the method was from 0 to 100 mg/L. The DOC of samples was calculated based on the difference between total carbon and inorganic carbon concentrations. All measurements were conducted in triplicate.

#### 2.5.3. Spectroscopic Analysis

Fourier transform infrared (FTIR) spectroscopy.

The samples were stored in a desiccator with the presence of allochronic silica gel before measurement. The FTIR spectra of samples were obtained by a Thermo Scientific Nicolet iS50 FT-IR (Thermo Fisher, St. Waltham, MA, USA) with a horizontal attenuated total reflectance (ATR) accessory. The FTIR spectra of the membrane samples were acquired by using the FTIR spectrometer with a horizontal attenuated total reflection device. The instrument was set to acquire spectra at 4 cm^−1^ resolution, within a spectral range of 4000–400 cm^−1^ and with 32 scans.

Excitation–emission matrix (EEM) fluorescence spectroscopy.

The EEM spectra of samples were determined using a spectrometer (HORIBA Aqualog, Tokyo, Japan), which enables simultaneous fluorescence and absorbance measurements with matched optical bandpass resolution. For EEM fluorescence measurements, EEM spectra were collected with subsequent scanning excitation spectra at 1 nm increments from 240 to 800 nm by varying the emission wavelength at 1.17 nm increments from 250 to 800 nm, and the fluorometer’s response to a blank solution was subtracted from the EEM spectra recorded for samples. A blank solution was used for preparation with ultrapure water. The EEM spectra of each sample were corrected for the inner filter effect according to absorbance data.

## 3. Results and Discussion

### 3.1. Characteristics of DOM Fractions Derived from EOM and IOM

#### 3.1.1. Hydrophobicity Distribution of Different Fractions

As shown in Table S1, the DOM derived from EOM and IOM was fractionated into three components: HPI, TPI, and HPO. For any particular group of EOM or IOM, the mass fractions obeyed the following order: HPO > HPI > TPI. It can be observed that 33.7% of DOC belonged to HPI, and nearly half of DOC was HPO, while only 10% of DOC belonged to TPI, which suggests that the dominant fraction in IOM was HPO. But HPI, HPO, and TPI accounted for 33.4%, 37.0%, and 26.6% of EOM. These results were in agreement with Zhang et al. [20] but different from those of Qu et al. [18]. Of note, the complex nature of the water matrix (i.e., the BG11 medium) in EOM may cause a shift in hydrophilicity. In terms of DOC, the HPI and HPO of IOM accounted for 33.7% and 56.3% respectively. TPI accounted for only a small proportion (10.0%). In our work, the proportion of HPO in AOM was higher than that in the results (35.17 ± 3.01%) of Zhao et al. [13] but lower than the results (44–88%) of Zhang et al. [21], which is likely because HPO can exhibit hydrophobicity due to its aromatic structure (high SUVA), as well as hydrophilicity due to its ionizable acid functional groups [22]. The predominance of the HPO in IOM can be attributed to the high abundance of aromatic proteins, cellular metabolites, and membrane-associated organic compounds released during cell lysis. These components contain abundant aromatic rings, carboxyl groups, and hydrophobic side chains, resulting in strong hydrophobicity. In contrast, EOM contains more soluble microbial products, including extracellular polysaccharides and small-molecule organic acids, which increase the proportion of HPI and TPI.

**Figure 1:**
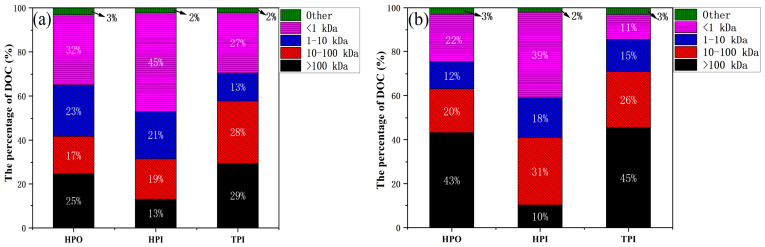
Molecular weight distribution of hydrophobicity fraction for EOM (**a**) and IOM (**b**).

To further investigate the effect of MW on the hydrophobicity of EOM and IOM, the DOM derived from three different fractions was further divided into four MW groups: >100, 10–100, 10–1, and <1 kDa. Figure 1 shows the DOM fractions with different hydrophobicities for the HPO, HPI, and TPI of EOM. The <1 kDa fraction contributed 68.9%, 57.6%, and 54.0% of DOC, accounting for a higher proportion than the other fractions. For the HPO, HPI, and TPI of IOM, the <1 kDa fraction contributed 64.1%, 47.9%, and 29.1% DOC, respectively. It is noteworthy that in EOM and IOM, the content of <100 kDa DOM in HPO, HPI and TPI is greater than 50%. Therefore, there is an urgent need to focus on the small molecules in HPO, and these fractions were shown to be less effective in membrane filtration processes for removal. The high proportion of low-molecular-weight fractions (<1 kDa) in all hydrophobicity components indicates that most AOM components are small molecules, which are difficult to retain using ultrafiltration membranes alone. This explains why conventional membrane processes show limited removal efficiency for AOM-associated DBP precursors.

#### 3.1.2. EEM Characteristics of Different Fractions

EEMs allow for the identification of humic- and protein-like substances and provide important information for studying the properties of AOM. As shown in Figure S2, there are three common fluorescence regions in the EEM spectra of AOM, each representing a specific component of DOM, including a protein-like region (Ex: 250–300 nm; Em: 300–390 nm), a fulvic acid-like region (Ex: 250–300 nm; Em: 400–470 nm), and a humic acid-like region (Ex: 300–400 nm; Em: 400–470 nm), as shown in Figure S2. Region I is often referred to as the protein-like peak and is thought to consist mainly of protein-like and amino acid fluorescence peaks. Regions II and III, which may be referred to as humic-like, are mainly fulvic acid-like and humic acid-like [23].

The EEM fluorescence spectra of the different hydrophobic components of the AOM are shown in Figure S3. The intensity of the fluorescence region (FRI) was used to reflect the content of organic matter, with protein-like and humic-like substances dominating the EOM and IOM. When the EOM solution was treated with DAX-8 resin and DAX-4 resin, only 54.2% and 65.7% in II and III were present in the HPI obtained. In contrast, in the eluate (HPO) of the DAX-8 resin, 78.2% of these were present in I and 49.2% and 21.5% in II and III. Meanwhile, only a slight amount of fulvic acid-like peaks, about 13.9%, were present in the eluate (TPI) obtained after the subsequent treatment with the DAX-4 resin. These results indicate that there are a few humic-like substances, but most are protein-like substances in the HPO of EOM. These results are highly consistent with those reported in [24,25]. And the majority of the humic-like substances were HPI, with a small amount of fulvic acid present in TPI for EOM. Figure S3 shows that when the IOM solution was treated with the DAX-8 resin and DAX-4 resin, only a small amount of organic substances in the humic-like region of IOM was in HPI, accounting for about 25% of the II region. The FRI of the I, II, and III regions in HPO accounts for about 91.6%, 40.6%, and 45.0%. These results indicated that most of the humic- and protein-like substances in IOM were HPO. A small proportion of the humic substances was also HPI.

**Figure 2:**
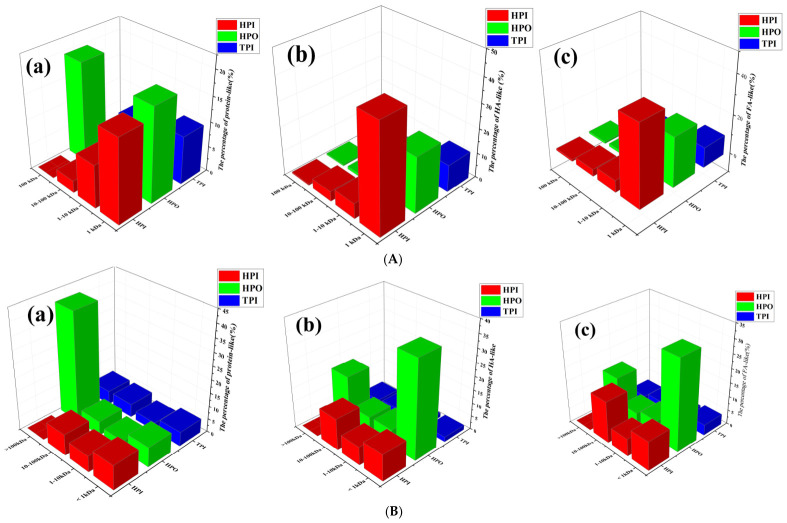
The EEM spectra for different MWs from hydrophobicity fractions of (**A**) EOM and (**B**) IOM.

The EEM spectra for different MW fractions of DOM with different hydrophobicities derived from AOM are shown in Figure 2. Protein-like and humic-like substances dominate in EOM and IOM. As shown in Figure 2A, for EOM, protein-like fractions were mostly distributed in HPO, where both MW > 100 kDa and MW< 1 kDa fractions were distributed, each accounting for about 20% of protein-like fractions, and <1 kDa HPI also contained about 15% of protein-like fractions. And humic acid-like (43%) and fulvic acid-like fractions (40%) were mainly distributed in <1 kDa HPI. Qu et al. [24] also demonstrated that most of the proteins for EOM were distributed in the high-MW and low-MW fractions, while the small molecules are low-MW acids and humic-like substances. As shown in Figure 2B, for IOM, protein-like fractions were mostly distributed in HPO with an MW > 100 kDa, accounting for about 41% of all proteins. The high fluorescence intensity of protein-like substances in high-MW HPO fractions (>100 kDa) confirms that macromolecular proteins are the main hydrophobic components in both EOM and IOM. These proteins contain aromatic amino acids such as tryptophan and tyrosine, which contribute to strong fluorescence signals and high DBP formation potential. And humic-like substances were also distributed in <1 kDa HPO for IOM, while fulvic acid-like and humic acid-like substances occupied 36% and 31% in <1 kDa HPO, respectively. Many scholars believe that macromolecules are mainly proteins, but conclusions about the hydrophilicity of proteins for IOM are significantly variable. These opposite results may come from differences in the cultivation or particular structure of the protein [23,26]. These results imply that the removal of humic-like organic matter from AOM by MWCO > 1 kDa filter membranes is extremely low, the humic acid-like and fulvic acid-like fractions in <1 kDa HPI of EOM were higher than other fractions, the humic acid-like and fulvic acid-like fractions in <1 kDa HPO of IOM were higher than other fractions, and these HPO components consisting of hydrophobic and transitional acids can be repelled by negatively charged membranes, implying that EOM may be more readily adsorbed to membranes than IOM. The enrichment in humic-like and fulvic-like substances in low-MW (<1 kDa) fractions indicates that these oxidized aromatic components are mainly small molecules. Since they carry negative charges and have small molecular sizes, they are difficult to adsorb or retain on UF membranes, which explains their low removal rate during membrane filtration.

### 3.2. The Removal of AOM

#### 3.2.1. DOC

**Figure 3:**
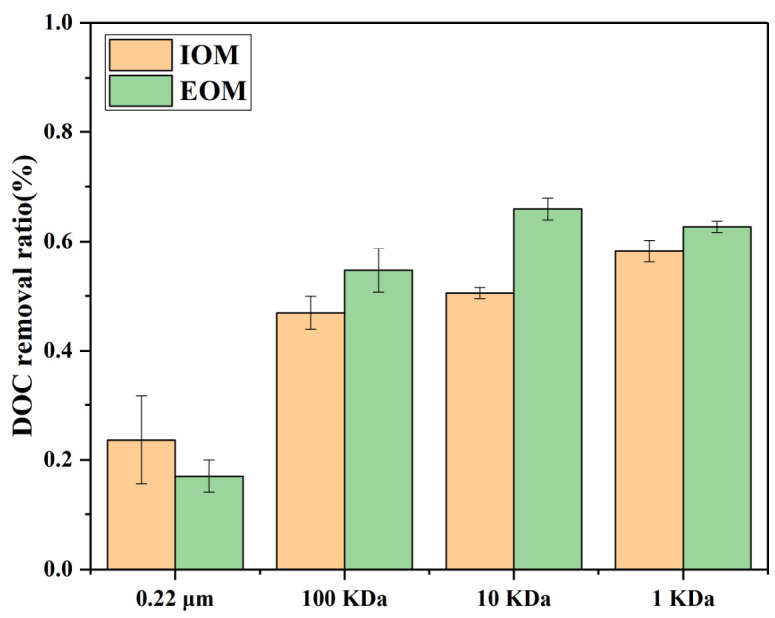
The removal of EOM and IOM by different types of membranes.

The removal efficiencies (represented by DOC values) of AOM by the UF process are shown in Figure 3. The results indicated that the DOC removal of AOM could increase with the pore size of the membrane decreasing. The removal efficiency of EOM and IOM was approximately 11% and 9% via the 0.45 µm membrane. And the difference in removal efficiency via the 0.45 and 0.22 µm filter membranes was not significant, while the UF membrane with an MWCO < 100 kDa could remove about 51% for IOM, and the removal efficiency of EOM was slightly better than that of IOM, accounting for 66%. The >100 kDa fraction of EOM was less than the removal fraction of the 100 kDa UF membrane, which is because the organic matter was adsorbed on the filter membrane [25]. Henderson et al. [27] reported that high-MW organic components (>100 kDa) were mainly seen for *Microcystis aeruginosa*, and this result confirms this. There was little difference in the ability of the UF membrane (100~1 KDa) to remove organic matter, which indicates that the removal of organic substances with an MW > 100 kDa was about 50%, while that of organic substances with an MW < 1 KDa was about 34%. And the contribution of low-MW organic substances < 1 kDa of IOM in DOC is higher than that of EOM. DOC removal efficiency increased significantly as membrane pore size decreased, because large-MW AOM components (>100 kDa) were effectively sieved. However, the removal of low-MW fractions (<1 kDa) remained poor, which is consistent with the fluorescence results showing that humic-like substances are mainly small molecules. This indicates that membrane filtration alone cannot fully control DBP precursors derived from small-molecule AOM.

#### 3.2.2. EEM Characteristics

**Figure 4:**
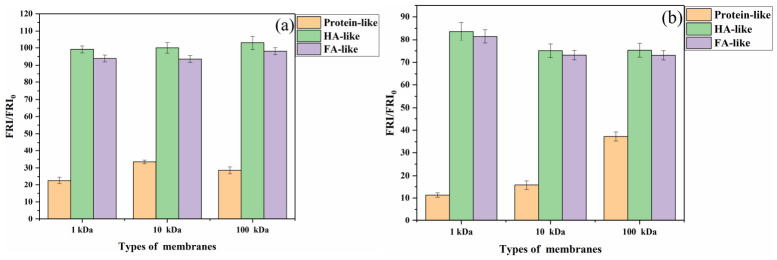
The removal of FRI in the EEM spectra from (**a**) EOM and (**b**) IOM by different MWCO membranes.

The EEM spectra of AOM at different MWs are shown in Figure S4. When the EOM was filtered through the 100 kDa MWCO membrane, the intensity of the A peak decreased significantly. However, the intensity of the peaks for humic-like substances (B and C) did not decrease as dramatically as it did for protein-like substances after, even when using a 1 kDa membrane. This is shown in Figure 4. The fluorescence region (FRI) of MW > 100 kDa occupied 71.5% of the total protein-like region for EOM. And the FRI of MW < 1 kDa occupied 97.3% of the total humic-like region. This indicates that humic-like substances are mostly low-molecular-weight (MW < 1 kDa) components in EOM and that the high-molecular-weight (MW > 100 kDa) organic matter contains a large number of protein-like substances. The humic-like substances of IOM were also of low molecular weight (MW < 1 kDa), and the high-molecular-weight (MW > 100 kDa) organic matter contains a large number of protein-like species. A total of 62.6% of the total protein-like region was occupied by an FRI of >100 kDa. But there were still some protein-like species present at 10~100 kDa for IOM, particularly phycocyanin and chlorophylls. The humic-like substances of IOM were similarly distributed to those of EOM, being mostly low-molecular-weight (<1 kDa) components, for which the UF process was also extremely ineffective, but there was still a slight change after the UF process with different MWCOs. When 100, 10, and 1 kDa membranes were selected, the FRI of region A of IOM and EOM after the UF process varied significantly, but there was no significant difference in the FRI of regions B and C of IOM after the UF process. After UF treatment, protein-like fluorescence decreased significantly, while humic-like fluorescence remained nearly unchanged. This mechanistic difference reveals that membrane fouling is mainly caused by macromolecular proteins and polysaccharides, whereas humic substances pass through the membrane due to their small size and high hydrophilicity.

#### 3.2.3. FTIR Characteristics

**Figure 5:**
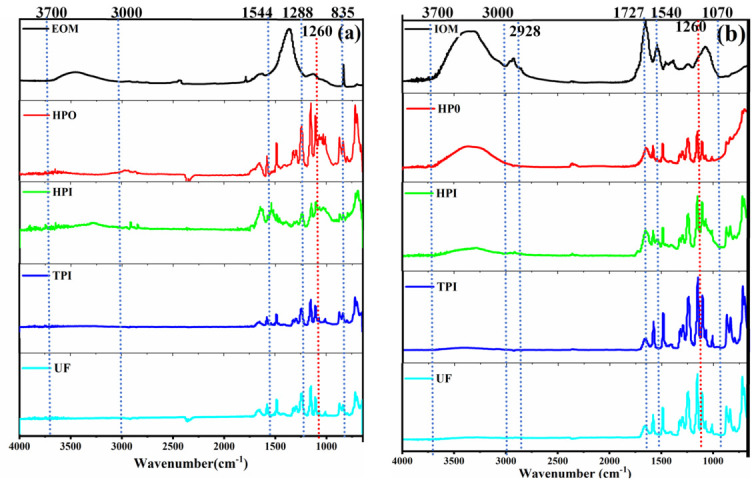
FTIR analysis of membranes after filtering (**a**) EOM and (**b**) IOM.

As shown in Figure 5, several peaks were found in the membranes filtered for different hydrophobic fractions of EOM and IOM. In our previous studies, the major bioactive components of EOM and IOM could be identified through FT-IR spectra: the characteristic bands for proteins were located at 3700–3000 cm^−1^, 1655 cm^−1^, and 1500–1300 cm^−1^; the characteristic bands for oils were at 3000–2800 cm^−1^, 1500–1300 cm^−1^, and 1000–1100 cm^−1^; and the characteristic bands for polysaccharides were at 1500–1300 cm^−1^, 950–1125 cm^−1^, and 835 cm^−1^. Detailed spectral characteristics of other FT-IR spectra are reported in previous studies [28]. The peaks at 1544~1288 cm^−1^ for the HPO and HPI of EOM disappeared after filtration, which was related to the humic component [29]. A band at ~1260 cm^−1^ was found for the HPO and HPI of EOM compared to the clean membrane, and this peak related to the stretching vibrations of the CO, C-N, S=O, C-H, C-O-H, and O=C-H of COOH, which point to polysaccharides and amino acids [30]. For IOM, a band at ~1260 cm^−1^ was also added to the clean membrane after membrane filtration for HPI. And the 1727 cm^−1^ related to the protein component was also noticed significantly for HPI [31]. In conclusion, the adsorbents on the membrane were mainly proteins or polysaccharides, and the humic component was largely not deposited on top of the membrane. The UF membrane was the most effective in removing macromolecular organic matter, and the proteins and polysaccharides were deposited on the membrane. The subsequent measurements of polysaccharides revealed that the polysaccharide content of the filtrate was extremely low and almost undetectable. The AOM adsorbed on the UF membrane mostly comprised protein- and polysaccharide-like substances and consisted of high-molecular-weight fractions; these findings could be further confirmed by another study [22]. FTIR peaks at ~1260 cm^−1^ and 1726 cm^−1^ confirm that proteins and polysaccharides are the dominant membrane foulants. The absence of humic-related adsorption peaks indicates that humic substances do not contribute to membrane fouling. This mechanism is critical for optimizing membrane cleaning and anti-fouling strategies

### 3.3. DBP Characteristics During Chlorination

**Figure 6:**
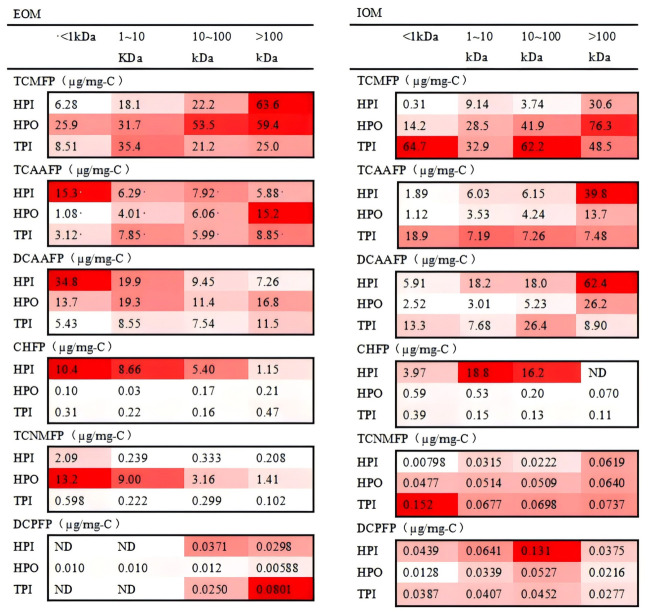
The DBP formation potential of different hydrophobicity fractions with different molecular weights in EOM and IOM.

**Figure 7:**
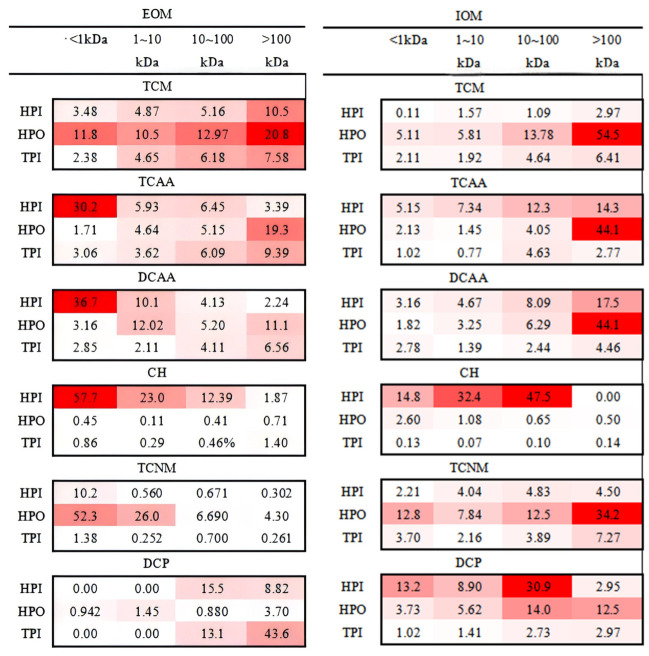
The contribution of DBPs for different hydrophobicity fractions with different molecular weights in EOM and IOM.

To eliminate the concentration effect, DBPFP was normalized against DOC. The DBPFP results for different characteristic components of AOM are shown in Table S2 and Figure 6, partial chromatograms of samples are shown in Figure S5, and the contribution of different characteristic components of AOM to DBP are shown in Figure 7.

The DBPFP results for different hydrophobic fractions of AOM are shown in Table S2. The TCMFP of HPO for EOM was much higher than that of HPI and TPI, which showed a large amount of protein-like substances and some humic-like substances, but the TCMFP of HPO and TPI in IOM was much higher than that of HPI, indicating that there were other small molecules with low TCMFP for the HPI of IOM. The TCMFP of HPO for EOM and IOM reached 37.1 and 37.4 µg/mg-C, respectively. These results also verify that the HPO with abundant aromatic components is a favorable precursor for TCM in AOM. The highest TCAAFP (11.9 and 17.9 µg/mg-C) and DCAAFP (26.2 and 16.4 µg/mg-C) were found for the HPI of IOM and EOM, respectively. The DCAAFP and TCAAFP (23.8 and 9.08 µg/mg-C, respectively) generated by the HPO of EOM were similar to that of HPI in IOM. The protein-like and humic-like substances in HPI and HPO had different proportions, which implied that the DCAAFP and TCAAFP from protein-like and humic-like substances in IOM were closer. And the TCAAFP of HPI was higher than that of HPO for EOM, while the DCAAFP of HPO and TPI for EOM was similar, considering the distribution of the protein-like substances and humic-like substances in HPO, HPI, and TPI, which implied that the DCAAFP of protein-like substances and humic-like substances for EOM was similar, and the TCAAFP of humic-like substances was higher than that of others. The fraction of the highest CHFP and DCPFP for EOM (2.36 and 0.081 µg/mg-C) and IOM (6.77 and 0.075 µg/mg-C) was mainly from the HPI, which implied that humic acid-like fractions may be important CH and DCP precursors. The precursors of the highest TCNMFP (14.9 and 0.125 µg/mg-C) were HPO for EOM and TPI for IOM. Organic N compounds are known to react with chlorine to form TCNM. For example, Methylamine and aspartic acid reacted with chlorine to form TCNM. However, the TCNMFPs of organic N compounds differed significantly [32,33].

#### 3.3.1. TCM

For HPI, the TCMFP of HPI-derived fractions from EOM was low, but the TCMFP of the MW > 100 kDa fractions was extremely high, reaching 63.6 µg/mg-C. For HPO, the TCMFP decreased with decreasing MW ranging from 25.9 to 59.4 µg/mg C for EOM and 14.2 to 76.3 µg/mg C for IOM. This trend indicated that the TCMFP of protein-like substances was higher than that of humic-like substances for the HPO of EOM and IOM. The TCMFP of TPI has no obvious trend for EOM and IOM. The TCMFP of the TPI for EOM was lower than that of IOM, which was in the range of 8.51~35.4 µg/mg-C for EOM and 32.9~64.7 µg/mg-C for IOM. DBP yields were determined by the content of precursors and TCMFP. The contribution of each fraction to TCM can be determined by combining the percentage of DOC and TCMFP from precursors. As shown in Figure 7, >100 kDa HPO contributed the most to TCM for EOM, nearly 20%. Additionally, >100 kDa HPO contributed the most to TCM for IOM, nearly 54%. This was the reason why the 100 kDa membrane had a better control effect of TCM on IOM than EOM. The extremely high TCMFP in >100 kDa HPO fractions indicates that hydrophobic macromolecular proteins are the key precursors for TCM. Aromatic structures and amino groups in proteins are easily attacked by chlorine to form trichloromethane. The higher contribution in IOM suggests that cell lysis will greatly increase TCM formation risk, which should be avoided in actual water plants.

#### 3.3.2. HAAs

Figure 6 shows the HAAFP for the fractions of AOM. The results indicated that HAAFP increased with the decrease in MW for the HPI of EOM. Moreover, <1 kDa HPI is likely a favorable precursor to the formation of HAAs for EOM, which might be attributed to the presence of phenolic hydroxyl groups, as well as humic substances, aldehydes, ketones, amino acids and other related components that could easily react with Cl_2_ to produce HAAs [34,35]. This research found that EOM contains mainly more fatty acids and humic acid in the low-MW fraction (<1 kDa) of HPO. As for the HPI of IOM, HAAFP increased with an increase in MW, indicating that the large-MW fraction of HPI contains more components with phenolic hydroxyl groups for IOM, e.g., amino acids, proteins, and aldehydes. In addition, the highest TCAAFP and DCAAFP (15.3 and 34.8 µg/mg-C) were found in the MW < 1 kDa fraction of HPI for EOM, and the highest TCAAFP and DCAAFP (39.8 and 62.4 µg/mg-C) were seen in the MW > 100 kDa fraction of HPI for IOM. TCAAFP and DCAAFP showed similar trends in IOM, suggesting that TCAAFP and DCAAFP may have the same precursors for IOM. For HPO, TCAAFP in EOM increased with increasing MW, but there was no stable trend for DCAAFP in EOM, which may be related to the distribution of phenolics. The TCAAFP and DCAAFP of different MWs for TPI in EOM were similar to those in IOM, with TCAAFP and DCAAFP in the range of 3.12~8.85 and 5.43~11.5 µg/mg-C in EOM and 7.19~18.9 and 7.68~26.4 µg/mg-C in IOM. The contribution of each fraction in EOM and IOM was obtained by combining the HAAFP and percentage of precursors, as shown in Figure 7. For EOM, <1 kDa HPI contributed the most to TCAA and DCAA, about 30.0 and 36.1%, respectively. In contrast, for IOM, >100 kDa HPO contributed the most to TCAA and DCAA, both at 44.0%. HAA precursors in EOM are dominated by low-MW hydrophilic fractions, while in IOM they are dominated by high-MW hydrophobic fractions. This opposite trend is caused by different functional groups: EOM contains more aliphatic acids and hydroxyl groups, while IOM contains more protein and amino acid structures. This discovery provides a targeted basis for controlling HAAs under different algal growth states.

#### 3.3.3. CH

The CHFP was less than that of TCM, TCAA, and DCAA, but the levels were not low. As shown in Figure 6, HPI had the highest CHFP in EOM and IOM, and CHFP increased with a decrease in MW in the HPI of EOM, up to 10.4 µg/mg-C. In IOM, the CHFP was higher for the 1~10 kDa and 10~100 kDa fractions (18.8 and 16.2 µg/mg-C) of HPI than the others, respectively. The CHFP of the other fractions was less than 0.60 µg/mg-C. This difference may be due to differences in the distribution and primary mechanisms of EOM and IOM, and two possible formation mechanisms for CH are the reaction between aldehydes (formed when natural organic substances are oxidized) and chlorine and the reaction between amino acids and chlorine [36]. The HPI of IOM with an MW in the range of 100~10 kDa contributed the most to CH (47.55%), which contained complex components but less DOC content. The HPI of EOM with an MW < 1 kDa contributes a very high percentage (57%) to CH, which mainly consists of humic substances. CH is mainly formed from low-MW humic-like substances in EOM and medium-MW hydrophilic components in IOM. The formation pathway is related to the chlorination of aldehydes and amino acids. Since CH is a typical nitrogen-containing DBP, its formation potential reflects the contribution of dissolved organic nitrogen (DON) in AOM.

#### 3.3.4. TCNM

Comparing EOM and IOM, TCNMFP was higher in EOM than in IOM, and TCNMFP increased with the decrease in MW for HPO and HPI in EOM, and the highest TCNMFP (13.2 µg/mg-C) was generated by HPO in EOM. But the HPO of IOM shows the opposite; the highest TCNMFP (0.152 µg/mg-C) was generated by the 1 < kDa fraction of TPI in IOM, which might be mainly attributed to the DON concentrations and molecular structure in AOM. As shown in Figure 7, the <1 kDa HPO fraction contributed the most to TCNM formation in EOM, accounting for 52%. This suggests that humic acid-like substances may be the primary source of TCNM in EOM. In IOM, >100 kDa HPO made the largest contribution to TCNMFP with 34%, thus indicating that protein-like substances may be the main source of TCNM in IOM. TCNM is a typical nitrogenous DBP (N-DBP) and is closely related to organic nitrogen in proteins. In EOM, TCNM comes from small-molecule hydrophobic fractions; in IOM, it comes from macromolecular proteins. This difference reflects the different nitrogen-containing functional groups between EOM and IOM.

#### 3.3.5. DCP

As shown in Figure 6, the majority of DCPFP was generated by the MW > 10 kDa fraction in EOM, and the highest DCPFP (0.0801 µg/mg-C) was generated by >10 kDa TPI. In the HPO of IOM, DCPFP was essentially evenly distributed among the different MWs, indicating that different MWs in HPO were its precursors. In IOM, the highest DCPFP (0.131 µg/mg-C) was mainly formed by 10~100 kDa HPI. As shown in Figure 7, the highest contribution to DCP was found in >100 kDa TPI for EOM, about 44.2%, and in 10~100 kDa HPI for IOM, about 39.0%. The precursors of DCP were mainly concentrated in the range of 10~100 kDa, so the 1 kDa filter membrane was chosen to control it the best. DCP precursors are concentrated in 10–100 kDa fractions, which can be effectively controlled using 1 kDa UF membranes. This provides clear operational guidance for membrane selection in engineering.

## 4. Conclusions

This study systematically characterized algal organic matter (AOM) from Microcystis aeruginosa using two-dimensional fractionation by molecular weight (MW) and hydrophobicity and quantified its disinfection byproduct (DBP) formation. AOM was divided into 12 subfractions. Hydrophobic components (HPO) dominated in intracellular organic matter (IOM), while extracellular organic matter (EOM) showed a balanced distribution. Both EOM and IOM were mainly composed of low-MW (<1 kDa) and high-MW (>100 kDa) substances. Fluorescence analysis indicated that humic-like substances were concentrated in <1 kDa fractions, whereas protein-like substances dominated >100 kDa HPO. FTIR verified that proteins and polysaccharides were the main membrane foulants. Ultrafiltration efficiently removed high-MW protein-like precursors but not low-MW humic-like substances. The >100 kDa HPO in IOM was the primary precursor of TCM, TCAA, and DCAA, while the <1 kDa HPI in EOM dominated HAA and CH formation. These results identify key DBP precursors and provide a scientific basis for targeted membrane process optimization and DBP control in eutrophic source water treatment.

## Data Availability

The original contributions presented in this study are included in the article/Supplementary Material. Further inquiries can be directed to the corresponding author.

## Supplementary Materials

The following supporting information can be downloaded at https://www.mdpi.com/article/10.3390/toxics14040349/s1. Figure S1. Schematic diagram of organic resin fractionation process; Figure S2. The EEM characteristics of (a) IOM and (b) EOM; Figure S3. Excitation–emission matrix fluorescence spectroscopy of XAD resin fraction results for (a) EOM and (b) IOM; Figure S4. The EEM spectra for different MW fractions of DOM with different hydrophobicities derived from (a) IOM and (b) EOM; Figure S5: Chromatograms of DBPs for some samples; Table S1. Hydrophobicity distribution of AOM; Table S2. DBP formation potential by hydrophobic fractions of (a) EOM and (b) IOM.

## Abbreviations

The following abbreviations are used in this manuscript:

AOM	Algogenic organic matter
MW	Molecular weight
DBPs	Disinfection byproducts
EOM	Extracellular organic matter
IOM	Intracellular organic matter
HPO	Hydrophobic fraction
HPI	Hydrophilic fraction
TPI	Transphilic fraction
UF	Ultrafiltration
TCM	Trichloromethane
TCAA	Trichloroacetic acid
DCAA	Dichloroacetic acid
TCMFP	Trichloromethane formation potential
EEM	Excitation–emission matrix fluorescence spectroscopy
HAAs	Haloacetic acids
THMs	Trihalomethanes
DBPFP	DBP formation potential
HAAFP	Haloacetic acid formation potential
DOM	Dissolved organic matter
BV	Bed volumes
NaOH	Sodium hydroxide
DOC	Dissolved organic carbon
DCAN	Dichloroacetonitrile
TCAN	Trichloroacetonitrile
TCNM	Trichloronitromethane
DCP	1,1-dichloro-2-propanone
CH	Chloral hydrate
TCP	1,1,1-trichloro-2-propanone
FTIR	Fourier transform infrared
FRI	Fluorescence region integration
MWCO	Molecular weight cut-off
SUVA	Specific ultraviolet absorbance
DON	Dissolved organic nitrogen

## References

[1] Cheshire M.M., Mitch W.A. (2025). Algae-derived organic matter in drinking water sources and the formation of disinfection byproducts: A critical review. Curr. Opin. Environ. Sci. Health.

[2] Liu C., Liu H., Hu C., Chow A.T., Karanfil T. (2024). Molecular Alterations of Algal Organic Matter in Oxidation Processes: Implications to the Formation of Disinfection Products. ACS EST Water.

[3] Huang W., Zhang Y., Lv W., Yang H., Yuan Q., Zhou W. (2025). Investigation on the membrane fouling control and mechanism induced by IOM using heat-activated peroxydisulfate pre-oxidation. RSC Adv..

[4] Li L., Cheng S., Wang Z., Zhang W., Zhang X., Zhang H. (2025). A critical review of the contradictory roles of algal organic matter in microalgae coagulation-flocculation: Effects of composition, properties, and mechanisms. Water Res..

[5] Li K., Li S., Sun C., Huang T.L., Li G.B., Liang H. (2020). Membrane fouling in an integrated adsorption-UF system: Effects of NOM and adsorbent properties. Environ. Sci.-Water Res. Technol..

[6] Xie Y., Fang Y., Chen D., Wei J., Fan C., Zhu X., Liu H. (2025). Membrane Fouling Control and Treatment Performance Using Coagulation–Tubular Ceramic Membrane with Concentrate Recycling. Membranes.

[7] Thakur A.K., Sathyamurthy R., Velraj R., Lynch I., Saidur R., Pandey A.K., Sharshir S.W., Kabeel A.E., Hwang J.-Y., GaneshKumar P. (2021). Secondary transmission of SARS-CoV-2 through wastewater: Concerns and tactics for treatment to effectively control the pandemic. J. Environ. Manag..

[8] Braeken L., Ramaekers R., Zhang Y., Maes G., Bruggen B.V.d., Vandecasteele C. (2005). Influence of hydrophobicity on retention in nanofiltration of aqueous solutions containing organic compounds. J. Membr. Sci..

[9] Zhou S., Zhu S., Shao Y., Gao N. (2015). Characteristics of C-, N-DBPs formation from algal organic matter: Role of molecular weight fractions and impacts of pre-ozonation. Water Res..

[10] Hua L.-C., Lin J.-L., Chao S.-J., Huang C. (2018). Probing algogenic organic matter (AOM) by size-exclusion chromatography to predict AOM-derived disinfection by-product formation. Sci. Total Environ..

[11] Huang W., Chu H., Dong B. (2014). Understanding the fouling of algogenic organic matter in microfiltration using membrane–foulant interaction energy analysis: Effects of organic hydrophobicity. Colloids Surf. B Biointerfaces.

[12] Cheng Z., Lin Z., Chen X., Zhang X., Zhang H. (2025). Unraveling the mechanisms underlying AOM-induced deterioration of the settling performance of algal floc. Water Res..

[13] Zhao Z., Sun W., Ray A.K., Mao T., Ray M.B. (2020). Coagulation and disinfection by-products formation potential of extracellular and intracellular matter of algae and cyanobacteria. Chemosphere.

[14] Chon K., Cho J., Shon H.K. (2013). Advanced characterization of algogenic organic matter, bacterial organic matter, humic acids and fulvic acids. Water Sci. Technol..

[15] Zhang T., Ma H., Hong Z., Fu G., Zheng Y., Li Z., Cui F. (2022). Photo-Reactivity and Photo-Transformation of Algal Dissolved Organic Matter Unraveled by Optical Spectroscopy and High-Resolution Mass Spectrometry Analysis. Environ. Sci. Technol..

[16] Pivokonsky M., Safarikova J., Baresova M., Pivokonska L., Kopecka I. (2014). A comparison of the character of algal extracellular versus cellular organic matter produced by cyanobacterium, diatom and green alga. Water Res..

[17] Hua L.-C., Chao S.-J., Huang C. (2019). Fluorescent and molecular weight dependence of THM and HAA formation from intracellular algogenic organic matter (IOM). Water Res..

[18] Qu F., Liang H., Tian J., Yu H., Chen Z., Li G. (2012). Ultrafiltration (UF) membrane fouling caused by cyanobateria: Fouling effects of cells and extracellular organics matter (EOM). Desalination.

[19] Hua G., Reckhow D.A. (2007). Characterization of disinfection byproduct precursors based on hydrophobicity and molecular size. Environ. Sci. Technol..

[20] Zhang X., Fan L., Roddick F.A. (2013). Influence of the characteristics of soluble algal organic matter released from Microcystis aeruginosa on the fouling of a ceramic microfiltration membrane. J. Membr. Sci..

[21] Zhang W., Zhang W., Zhang X., Amendola P., Hu Q., Chen Y. (2013). Characterization of dissolved organic matters responsible for ultrafiltration membrane fouling in algal harvesting. Algal Res..

[22] Her N., Amy G., Chung J., Yoon J., Yoon Y. (2008). Characterizing dissolved organic matter and evaluating associated nanofiltration membrane fouling. Chemosphere.

[23] Chen W., Westerhoff P., Leenheer J.A., Booksh K. (2003). Fluorescence excitation−emission matrix regional integration to quantify spectra for dissolved organic matter. Environ. Sci. Technol..

[24] Qu F., Liang H., He J., Ma J., Wang Z., Yu H., Li G. (2012). Characterization of dissolved extracellular organic matter (dEOM) and bound extracellular organic matter (bEOM) of Microcystis aeruginosa and their impacts on UF membrane fouling. Water Res..

[25] Ly Q.V., Lee M.-H., Hur J. (2019). Using fluorescence surrogates to track algogenic dissolved organic matter (AOM) during growth and coagulation/flocculation processes of green algae. J. Environ. Sci..

[26] Hu Y., Wang Z., Li B., Cheng S., Yang P., Zhang H., Li S., Zhang X. (2025). Unraveling the mechanistic role of algogenic organic matter (AOM) in coagulation efficiency: How interspecific variations in AOM govern coagulant dosage, floc characteristics, and settling dynamics. Sep. Purif. Technol..

[27] Henderson R.K., Baker A., Parsons S.A., Jefferson B. (2008). Characterisation of algogenic organic matter extracted from cyanobacteria, green algae and diatoms. Water Res..

[28] Ma L., Peng F., Dong Q., Li H., Yang Z. (2022). Identification of the key biochemical component contributing to disinfection byproducts in chlorinating algogenic organic matter. Chemosphere.

[29] Mayers J.J., Flynn K.J., Shields R.J. (2013). Rapid determination of bulk microalgal biochemical composition by Fourier-Transform Infrared spectroscopy. Bioresour. Technol..

[30] Oliveira R.C., Hammer P., Guibal E., Taulemesse J.-M., Garcia O. (2014). Characterization of metal–biomass interactions in the lanthanum(III) biosorption on Sargassum sp. using SEM/EDX, FTIR, and XPS: Preliminary studies. Chem. Eng. J..

[31] Benning L.G., Phoenix V.R., Yee N., Konhauser K.O. (2004). The dynamics of cyanobacterial silicification: An infrared micro-spectroscopic investigation. Geochim. Cosmochim. Acta.

[32] Yang X., Fan C., Shang C., Zhao Q. (2010). Nitrogenous disinfection byproducts formation and nitrogen origin exploration during chloramination of nitrogenous organic compounds. Water Res..

[33] Hu J., Song H., Addison J.W., Karanfil T. (2010). Halonitromethane formation potentials in drinking waters. Water Res..

[34] Wang X.-X., Liu B.-M., Lu M.-F., Li Y.-P., Jiang Y.-Y., Zhao M.-X., Huang Z.-X., Pan Y., Miao H.-F., Ruan W.-Q. (2021). Characterization of algal organic matter as precursors for carbonaceous and nitrogenous disinfection byproducts formation: Comparison with natural organic matter. J. Environ. Manag..

[35] Huber S.A., Balz A., Abert M., Pronk W. (2011). Characterisation of aquatic humic and non-humic matter with size-exclusion chromatography—Organic carbon detection—Organic nitrogen detection (LC-OCD-OND). Water Res..

[36] Barrott L. (2004). Chloral hydrate: Formation and removal by drinking water treatment. J. Water Supply Res. Technol.-Aqua.

