# Supplementary material for: The Characteristics of AOM and Formation of DBPs: The Role of Molecular Weights and Hydrophobicity
Source: Toxics. 2026 Apr 21;14(4):349. doi: 10.3390/toxics14040349 (PMC13119584; doi:10.3390/toxics14040349)
Supplement: Supplementary file 1 [file toxics-14-00349-s001.zip › toxics-4227848-supplementary.pdf]

Supplementary information

The characteristic and DBPs formation of AOM: Role of molecular weights and hydrophobicity

Lingfei Ma [1]* Haipu Li[2] Zhaoguang Yang[2]

[1] School of Petroleum Engineering and Environment Engineering, Yanan University, Yanan 716000, PR China
[2] Center for Environment and Water Resources, College of Chemistry and Chemical Engineering, Central South University, Changsha 410083, PR China
* Correspondence: malingfei@yau.edu.cn;

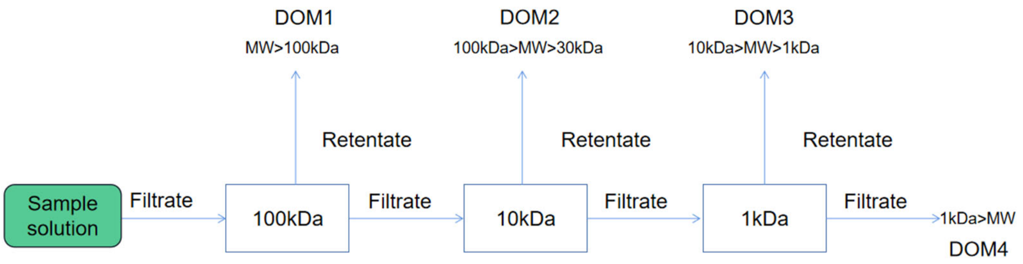

Figure S1. Schematic diagram of the organic resin fractionation process

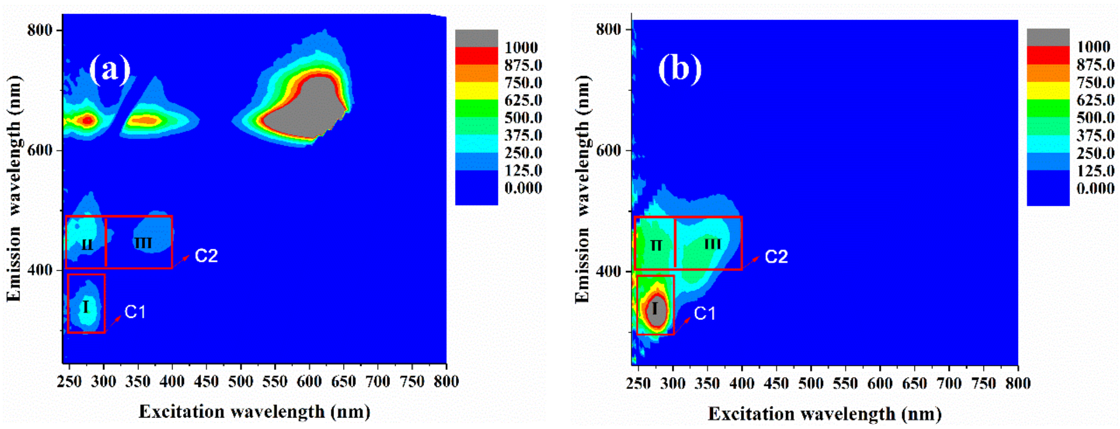

Figure S2. The EEM characteristic of (a) IOM and (b) EOM

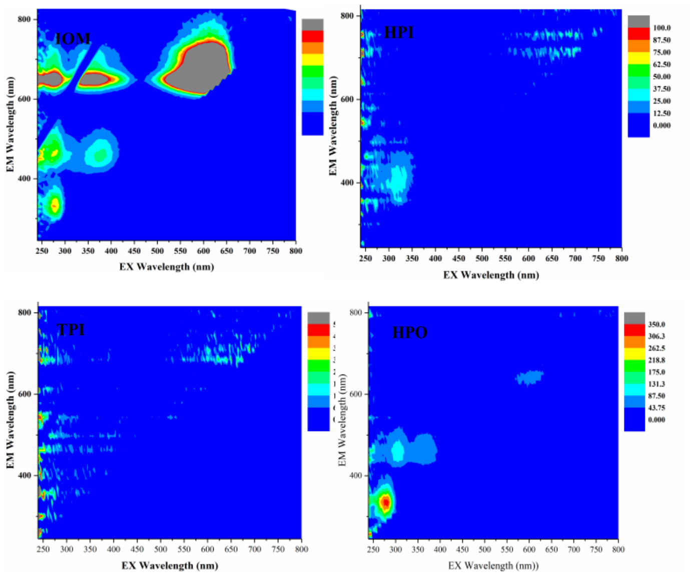

(a)

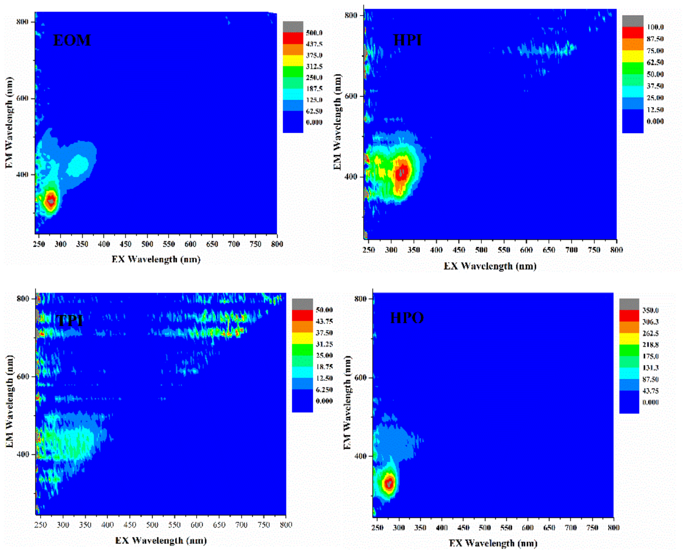

(b)

Figure S3. Excitation-emission matrix fluorescence spectroscopy of XAD resin fraction results for (a) EOM and (b) IOM

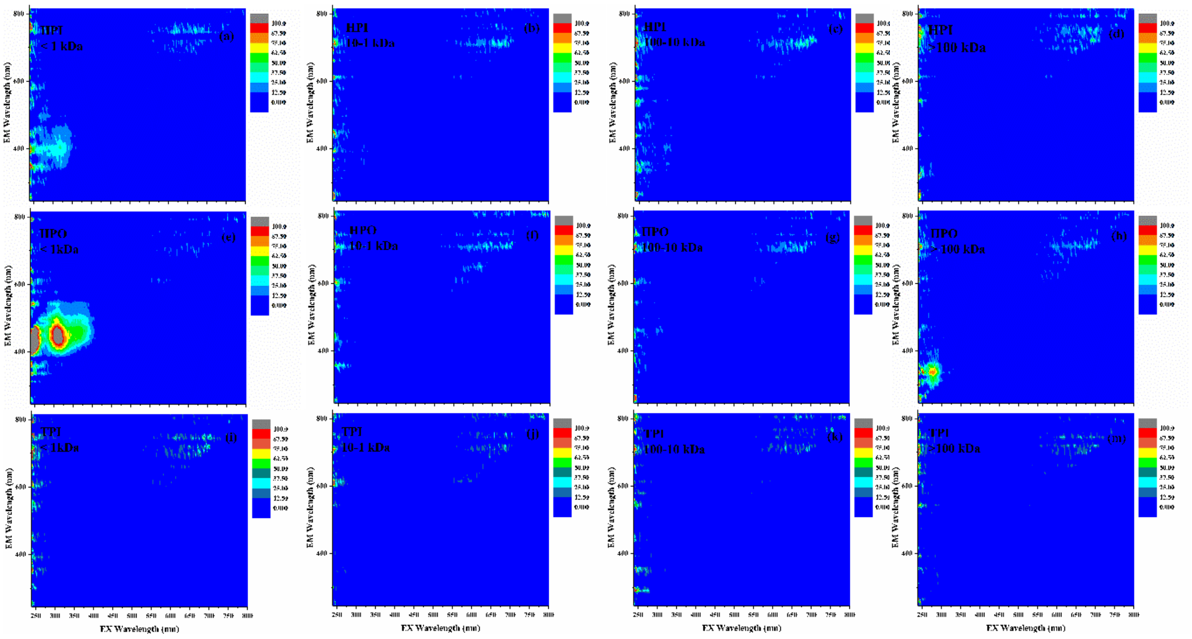

(a).

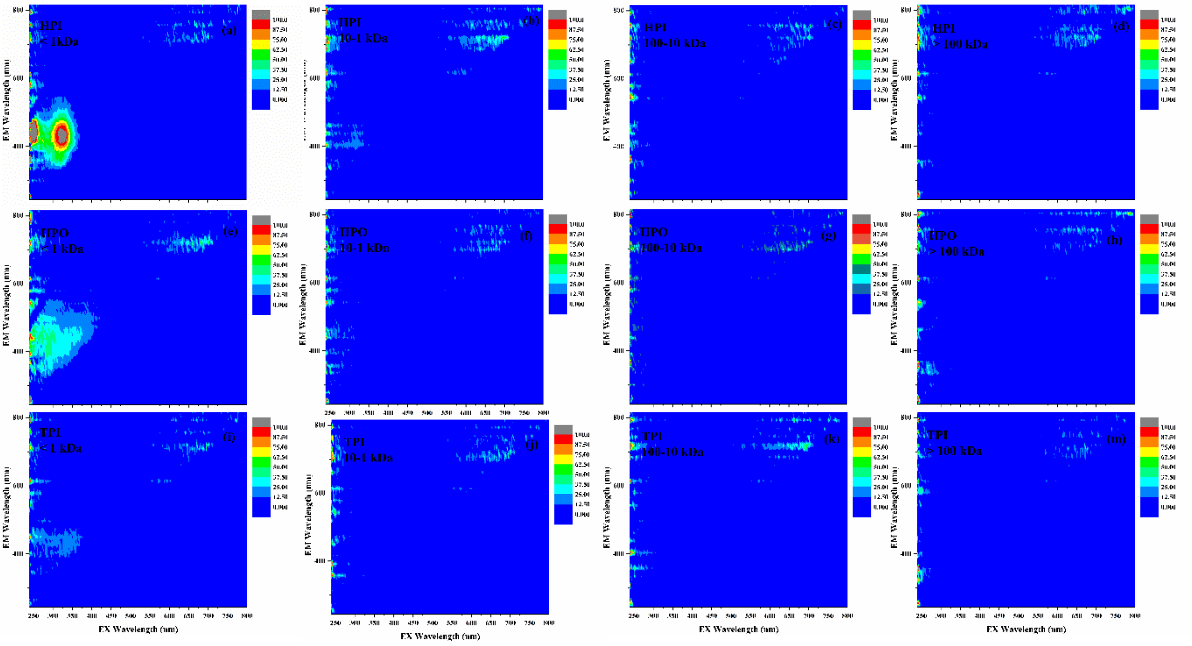

(b)

Figure S4. The EEM spectra for different MW fractions of DOM with different hydrophobicities derived from(a) IOM and (b)EOM

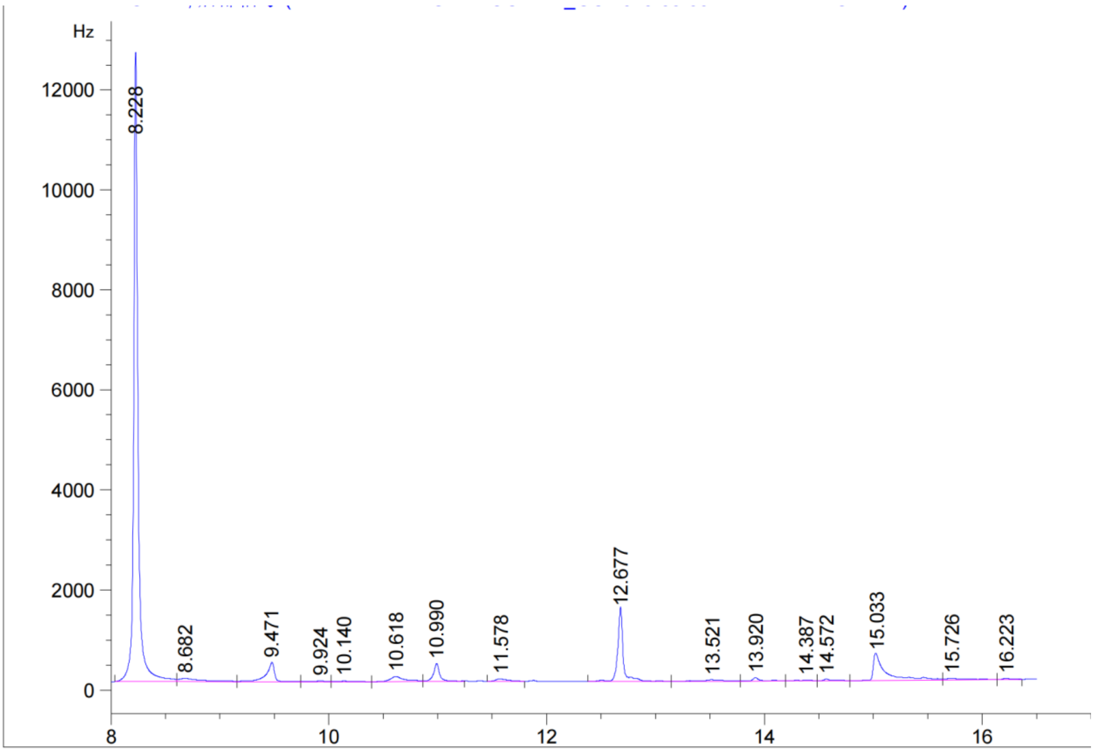
Hz
12000
10000
8000
6000
4000
2000
0
8.228
8.682
9.471
9.924
10.140
10.618
10.990
11.578
12.677
13.521
13.920
14.387
14.572
15.033
15.726
16.223
8
10
12
14
16

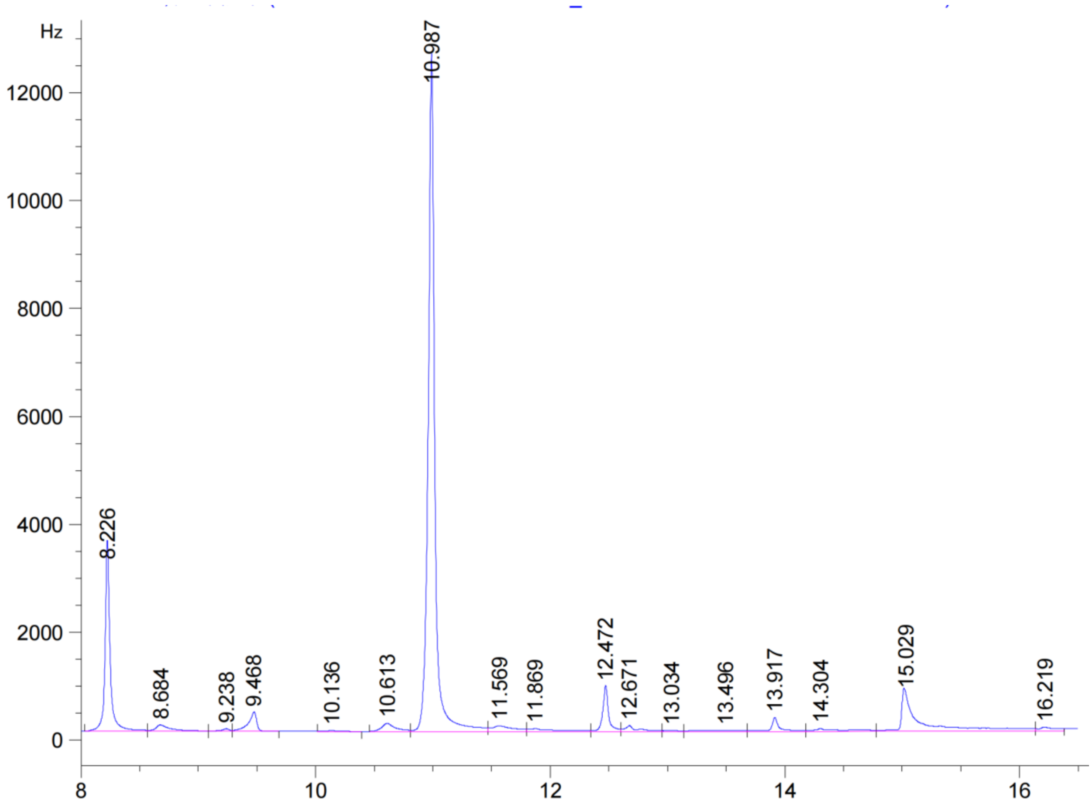
Hz
12000
10000
8000
6000
4000
2000
0
8.226
8.684
9.238
9.468
10.136
10.613
10.987
11.569
11.869
12.472
12.671
13.034
13.496
13.917
14.304
15.029
16.219
8
10
12
14
16

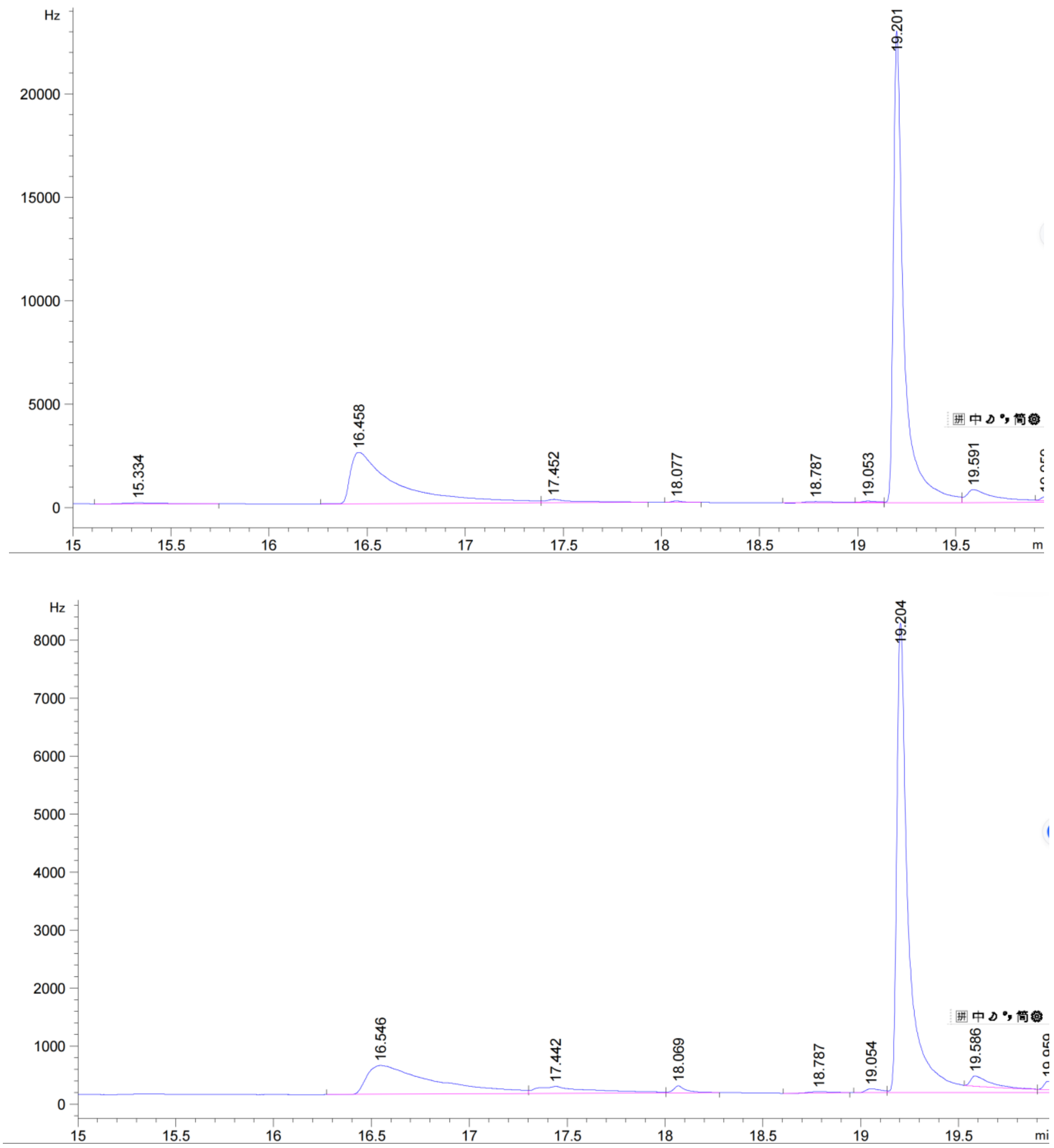

Figure S5. Chromatograms of DBPs for some samples

Table S1. Hydrophobicity distribution of AOM

Source water	HPO	HPI	TPI
EOM	37.0	33.4	26.6
IOM	56.3	33.7	10.0

Table S2. DBPs formation potential by hydrophobic fractions of EOM and IOM

	TCMFP		TCAAFP		DCAAFP		CHFP		TCNMFP		DCPFP	
	EOM	IOM	EOM	IOM	EOM	IOM	EOM	IOM	EOM	IOM	EOM	IOM
whole	36.9	39.2	10.3	8.41	18.7	17.5	0.125	3.10	0.706	0.071	/	0.00276
HPI	27.8	2.1	17.9	11.9	16.4	26.2	2.36	6.77	1.80	0.0136	0.0814	0.0750
HPO	37.1	37.4	5.62	9.08	15.2	23.8	ND	0.368	14.9	0.0244	0.00807	0.00655
TPI	22.3	42.4	5.08	6.87	5.65	8.52	0.0413	0.319	0.400	0.125	0.0218	0.0318
